# Multi-Omics Analysis of Fatty Alcohol Production in Engineered Yeasts *Saccharomyces cerevisiae* and *Yarrowia lipolytica*

**DOI:** 10.3389/fgene.2019.00747

**Published:** 2019-08-30

**Authors:** Jonathan Dahlin, Carina Holkenbrink, Eko Roy Marella, Guokun Wang, Ulf Liebal, Christian Lieven, Dieter Weber, Douglas McCloskey, Hong-Lei Wang, Birgitta E. Ebert, Markus J. Herrgård, Lars Mathias Blank, Irina Borodina

**Affiliations:** ^1^The Novo Nordisk Foundation Center for Biosustainability, Technical University of Denmark, Kongens Lyngby, Denmark; ^2^iAMB – Institute of Applied Microbiology, ABBt – Aachen Biology and Biotechnology, RWTH Aachen University, Aachen, Germany; ^3^ Department of Biology, Lund University, Lund, Sweden

**Keywords:** fatty alcohol, metabolome, ^13^C-fluxome, transcriptome, *Yarrowia lipolytica*, *Saccharomyces cerevisiae*

## Abstract

Fatty alcohols are widely used in various applications within a diverse set of industries, such as the soap and detergent industry, the personal care, and cosmetics industry, as well as the food industry. The total world production of fatty alcohols is over 2 million tons with approximately equal parts derived from fossil oil and from plant oils or animal fats. Due to the environmental impact of these production methods, there is an interest in alternative methods for fatty alcohol production *via* microbial fermentation using cheap renewable feedstocks. In this study, we aimed to obtain a better understanding of how fatty alcohol biosynthesis impacts the host organism, baker’s yeast *Saccharomyces cerevisiae* or oleaginous yeast *Yarrowia lipolytica*. Producing and non-producing strains were compared in growth and nitrogen-depletion cultivation phases. The multi-omics analysis included physiological characterization, transcriptome analysis by RNAseq, ^13^Cmetabolic flux analysis, and intracellular metabolomics. Both species accumulated fatty alcohols under nitrogen-depletion conditions but not during growth. The fatty alcohol–producing *Y. lipolytica* strain had a higher fatty alcohol production rate than an analogous *S. cerevisiae* strain. Nitrogen-depletion phase was associated with lower glucose uptake rates and a decrease in the intracellular concentration of acetyl–CoA in both yeast species, as well as increased organic acid secretion rates in *Y. lipolytica*. Expression of the fatty alcohol–producing enzyme fatty acyl–CoA reductase alleviated the growth defect caused by deletion of hexadecenal dehydrogenase encoding genes (*HFD1* and *HFD4*) in *Y. lipolytica*. RNAseq analysis showed that fatty alcohol production triggered a cell wall stress response in *S. cerevisiae*. RNAseq analysis also showed that both nitrogen-depletion and fatty alcohol production have substantial effects on the expression of transporter encoding genes in *Y. lipolytica*. In conclusion, through this multi-omics study, we uncovered some effects of fatty alcohol production on the host metabolism. This knowledge can be used as guidance for further strain improvement towards the production of fatty alcohols.

## Introduction

Fatty alcohols are used as detergents and surfactants in personal care products such as soaps, shampoos, or creams. The global market for fatty alcohols is estimated at 5 billion USD (Grand View Research, Inc. 2016). The major fraction of the fatty alcohols used today are derived from either crude oil or palm kernel oil, both feedstocks being non-sustainable on a long term (Shah et al., 2016). Alternatively, fatty alcohols can be produced from abundant renewable feedstocks *via* microbial fermentation.

Some marine bacteria can produce fatty alcohols naturally; however, the titers are low, and these organisms are not suitable for large-scale fermentation. Hence, several industrially applicable hosts have been engineered to produce fatty alcohols. *Escherichia coli* was engineered to produce 6.3 g/L total fatty alcohols (Liu et al., 2016) (bioreactor, complex media). Baker’s yeast *Saccharomyces cerevisiae* was engineered to achieve a titer of 6.0 g/L (d’Espaux et al., 2017) (bioreactor, complex media). Several oleaginous yeast species have also applied for fatty alcohol production. *Yarrowia lipolytica* has been engineered to produce 2.2 g/L (Xu et al., 2016) (bioreactor, minimal media), and *Lipomyces starkeyi* was engineered to produce 1.7 g/L (McNeil and Stuart 2018) (shake flask, minimal media). To this date, the highest reported titer of 8 g/L total alcohols was obtained with oleaginous yeast *Rhodosporidium toruloides* (Fillet et al., 2015) (bioreactor, complex media).

**Figure 1:**
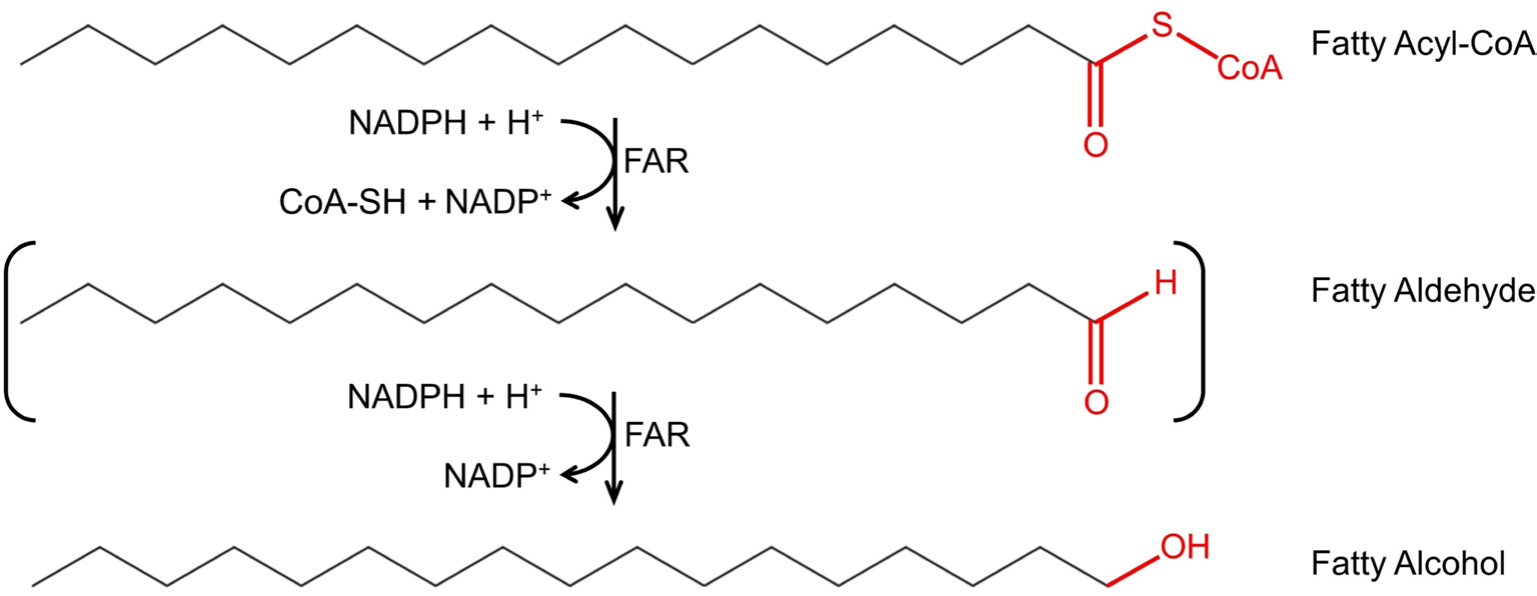
Fatty alcohol biosynthesis *via* fatty acyl–CoA reductase (*FAR*). FAR catalyzes the conversion of fatty acyl–CoAs to fatty alcohols with the fatty aldehyde as transient intermediate (indicated as brackets). The substrate depicted in the figure is hexadecanoyl-CoA; however, carbon chain length and saturation may vary.

Fatty alcohol biosynthesis is carried out in two enzymatic steps from fatty acyl–CoAs, key intermediates in membrane, and storage lipid biosynthesis. The two enzymatic steps can be carried out by two enzymes, an aldehyde-forming long-chain acyl–CoA reductase (ACR, EC 1.2.1.50), and an aldehyde reductase (AHR, EC 1.1.1.2), where ACR converts the fatty acyl–CoA into a fatty aldehyde, which in turn is converted by AHR into fatty alcohol. The two enzymatic steps can also be carried out by a single enzyme, an alcohol-forming fatty acyl–CoA reductase (FAR, EC 1.2.1.84), where FAR converts fatty acyl–CoA into fatty alcohol, with a fatty aldehyde as a transient intermediate. The conversion of fatty acyl–CoA into corresponding fatty alcohol requires two NADPH molecules (**Figure 1**).

The process performance parameters reported for fatty alcohols in the literature do not yet meet the requirements for industrial production of bulk chemicals. Further strain improvement and process optimization are required. A better understanding of the effect of fatty alcohol production on a cell factory can guide further strain improvement. In this study, we performed a multi-omics analysis comparing gene expression, fluxes, and intracellular metabolites’ concentrations in fatty alcohol–producing *S. cerevisiae* and *Y. lipolytica* strains.

## Materials and Methods

### Strains, Reagents, and Chemicals

*Escherichia coli DH5α* was used for manipulation of DNA during cloning. *S. cerevisiae CEN.PK113-7D* (*MATa URA3 HIS3 LEU2 TRP1 MAL2‐8^c^ SUC2*) was a gift from Dr. Peter Kötter (Goethe-Universität, Germany). *Y. lipolytica* GB20 (MATb, *ku70Δ, nugm-Htg2, ndh2i, lys11^−^, leu2^−^, ura3^−^*) was a gift from Prof. Volker Zickermann (Goethe-Universität, Germany). Cloning reagents were sourced according to the EasyClone-Markerfree (Jessop-Fabre et al., 2016) and EasyCloneYALI (Holkenbrink et al., 2018). All chemicals were acquired from Sigma-Aldrich unless otherwise specified. Nourseothricin was acquired from Jena Bioscience GmbH (Germany).

### Strain Construction

The *S. cerevisiae* strains were constructed using the EasyClone-MarkerFree toolbox (Jessop‐Fabre et al., 2016). The fatty alcohol degradation-deficient strain (ST6849) was constructed from CEN.PK113-7D by introducing a Cas9 plasmid pCfB2312, followed by a knock out of the *PEX10* and *HFD1* genes using the single guide RNA (sgRNA) plasmid and repair templates (synthesized DNA fragment) as described in **Supplementary Table S1**. The fatty alcohol–producing strain (ST6989) was constructed by expressing a total of four copies of the fatty acyl–CoA reductase from *Marinobacter algicola* (*malFAR*), codon-optimized for *Y. lipolytica* (sequence detailed in **Supplementary File S1**), in the degradation-deficient strain. Integration vectors containing two copies of *malFAR* were constructed, consisting of one copy of *malFAR* under the *TDH3* (*GPD*) promoter and one copy under the *TEF1* promoter, which were inserted into vectors pCfB3034 and pCfB3037 to generate vectors pCfB7082 and pCfB7083, respectively (**Supplementary Table S1**). The NotI-digested plasmids were inserted into integration sites X-3 and XI-5, using the double sgRNA plasmid pCfB5283.

The *Y. lipolytica* degradation-deficient strain (ST6770) was constructed from strain ST6276 (Borodina et al., 2018). ST6276 is derived from *Y. lipolytica* strain GB20 by deleting genes *FAO1*, *HFD1*, *HFD4*, and *PEX10*. The leucine and uracil auxotrophies were closed by integrating an expression cassette containing *URA3* and *LEU2* using vector pCfB7093 and selecting for growth on SC-Ura-Leu agar plates. The lysine auxotrophy was closed by restoring the native sequence of the homocitrate synthase gene (YALI1_F38776g) using DNA fragment BB2251 and selecting for growth on SC-Lys agar plates. The fatty alcohol–producing strain (ST6987) was constructed by expressing a total of four copies of the *malFAR*, codon-optimized for *Y. lipolytica*, in the degradation-deficient strain ST6770. A two-gene construct, consisting of one copy of *malFAR* under the *GPD* promoter and one copy under the *TEF*-intron promoter, were inserted into vector pCfB4796 and pCfB4784 to generate plasmid pCfB7091 and pCfB7092, respectively (**Supplementary Table S1**). The NotI-digested plasmids were inserted at both integration sites D-1 and F-2.

The promoter and coding sequences of all vectors were verified using Sanger sequencing provided by Eurofins Genomics, and integration was verified by colony PCR.

### Cultivation

Unless otherwise specified, all cultures were grown in baffled shake flasks equipped with caps containing air-permeable membranes, incubated at 30°C at 250 rotations per minute (RPM) in a MaxQ 8000 Orbital Shaker (Thermo Fisher). Pre-cultures were grown on minimal media until the late exponential phase, and subsequently washed and resuspended in either fresh minimal media or nitrogen-depleted media for growth phase or nitrogen-depleted stationary phase studies respectively. For the pre-cultures, the media contained 20 g/L glucose, 5 g/L ammonium sulfate, 12 g/L potassium phosphate (pH 6.0), 3.4 g/L yeast nitrogen base (YNB, w/o amino acids, w/o ammonium sulfate), and 1% YPD. Pre-cultures were centrifuged at 5,000 × g for 5 min at room temperature and washed with an equal volume of sterile water; cells were centrifuged again at 5,000 × g for 5 min at room temperature and resuspended to OD_600_ 10 in nitrogen-depleted media. For nitrogen-depleted stationary phase cultures, the resuspended cells were used as the starting point. For growth phase cultures, the resuspended cells were inoculated into growth phase media at OD_600_ 0.03. For the growth phase cultivations, mineral salt medium was used, containing 20 g/L glucose, 5 g/L ammonium sulfate, 12 g/L potassium phosphate (pH 6.0), and 3.4 g/L YNB (w/o amino acids, w/o ammonium sulfate). For the nitrogen-depleted stationary phase cultivations, the media contained 20 g/L glucose, 12 g/L potassium phosphate (pH 6.0), and 3.4 g/L YNB (w/o amino acids, w/o ammonium sulfate). For the fatty alcohol degradation test, cells were cultivated in triplicates in 12-ml round bottom glass vials filled with 2-ml media at 30°C at 250 RPM in a MaxQ 8000 Orbital Shaker (Thermo Fisher). The cultivation medium contained 500 mg/L hexadecanol, 500 mg/L octadecanol, 20 g/L ethanol, 2 g/L glucose, 1.7 g/L YNB w/o amino acids and ammonium sulfate, 12 g/L potassium phosphate (pH 6.0), and 5 g/L ammonium sulfate. Biological triplicates were done for each strain and condition.

### HPLC Analysis

Five hundred-microliter samples were taken at each sampling point, centrifuged in Eppendorf tubes at 5,000 × g at 4°C for 5 min, and supernatants were stored at −20°C until analysis. Supernatants were analyzed for the presence of ethanol, glucose, glycerol, and acetate using HPLC UltiMate 3000 (Thermo Fisher) with Aminex HPX87H ion exclusion column. Samples were run for 30 min at 0.600 ml/min at 60°C using 5 mM H2SO4 as eluent. Compounds were detected using a Dionex RI101 and DAD-3000 detectors (Dionex) for RI and UV detection, respectively.

### Liquid Chromatography-Mass Spectrometry (LC-MS) Analysis

For *Y. lipolytica* cultures, supernatant samples from section *HPLC Analysis* were also analyzed for the presence of tricarboxylic acid (TCA) cycle–derived organic acids (malic acid, succinic acid, citric acid, isocitric acid, pyruvic acid, αketoglutaric acid, and fumaric acid). LC-MS data were collected on the EVOQ Elite Triple Quadrupole Mass Spectrometer system coupled with an Advance UHPLC pump (Bruker, Fremont, CA). Samples were held in the CTC HTS PAL autosampler at a temperature of 5.0°C during the analysis. 1-μl injections of the sample were made onto a Waters ACQUITY HSS T3 C18 UHPLC column, with a 1.8-μm particle size, 2.1 mm i.d., and 100 mm long. The column was maintained at 30.0°C. The solvent system used was solvent A (MilliQ water with 0.1% formic acid) and solvent B (acetonitrile with 0.1% formic acid). The flow rate was 0.400 ml/min with an initial solvent composition of %A = 100 and %B = 0 held until 0.50 min; the solvent composition was then changed following a linear gradient until it reached %A = 5.0 and %B = 95.0 at 1.00 min. This was held until 1.79 min when the solvent was returned to the initial conditions, and the column was re-equilibrated until 4.00 min. The column eluent flowed directly into the heated ESI probe of the MS, which was held at 250°C and a voltage of 2,500 V. MRM data was collected in negative ion mode; the target masses are shown in **Supplementary Table S2**. The other MS settings were as follows: sheath gas flow rate of 50 units, nebulizer gas flow rate of 50 units, cone gas flow rate of 20 units, cone temp was 350°C, and collision gas pressure 1 mTorr.

### Cell Dry Weight

The cell dry weight (CDW) was determined by pre-weighing dried 0.45-µm cellulose nitrate membrane filters (VWR), filtering 10-ml culture across the membrane, washing with 10 ml water, drying at 60°C, and weighing the dried filter with biomass. The OD/CDW ratio was determined to be constant for all the strains throughout all the cultivations, and for the cultures throughout this study, the biomass was estimated by measuring the OD_600_ using a NanoPhotometer Pearl (Implen) and calculating the biomass dry weight using conversion factors of 0.12 (g/L)/OD or 0.14 (g/L)/OD for *S. cerevisiae* and *Y. lipolytica*, respectively.

### Fatty Alcohol Extraction

Fatty alcohols were analyzed by collecting 1 ml of culture broth into a 4-ml glass vial and adding 10-µl internal standard (2 g/L of methyl cis-10-heptadecanoate dissolved in 100% ethanol). Samples were vortexed for 3 s and frozen at 80°C until further processing. To perform the extraction, samples were freeze-dried for 2–3 days at −30°C under vacuum in a freeze-drying system (Labconco). 1 ml 2:1 chloroform:methanol mixture was added to each freeze-dried sample to disrupt the cells, vortexed for 15 min in a DVX-2500 multi-tube vortexer (VWR), and left at room temperature for 4 h. The solvents were subsequently evaporated under a nitrogen stream. 1 ml hexane was added to the sample vials, vortexed for 15 min in a DVX-2500 multi-tube vortexer (VWR), and incubated at room temperature overnight. Samples were transferred to new vials and stored at −20°C until analyzed.

The analysis was carried out on a GC-MS using an INNOWax column (30 m × 0.25 mm × 0.25 μm) with helium as carrier gas. The injector was set to splitless mode at 250>A°C; the oven temperature was set to 80°C for 1 min, increased at a rate of 10°C/min to 210°C, followed by a hold at 210°C for 15 min, increased at a rate of 10°C/min to 230°C followed by a hold at 230°C for 20 min. The GC-MS was operated in electron impact mode (70 eV), scanning at the range 30–400 m/z. Compounds were quantified relative to the internal standard (methyl cis-10-heptadecenoate).

### Fatty Alcohol Degradation

Fatty alcohol degradation was estimated by cultivating cells in media containing fatty alcohols. Strains used were CEN.PK113-7D (*S. cerevisiae* reference), ST6849 (*S. cerevisiae pex10Δ, hfd1Δ*), W29 (*Y. lipolytica* reference), and ST6770 (*Y. lipolytica pex10Δ, fao1Δ, hfd1Δ, hfd4Δ*) (**Table 1**). The negative controls did not contain any cells. Cultures were inoculated to a starting OD_600_ of 1.0 and incubated for 96 h. The whole cultivation tube containing 2 ml culture was taken as sample and processed for fatty alcohol quantification according to the previous description (*Cell Dry Weight*), but with double volumes to account for the increased sample size. The detection of slightly lower levels of hexadecanol and slightly higher levels of octadecanol than expected is likely due to an experimental error, which is rather high when working with hydrophobic substances in small volumes. Upon addition of fatty alcohols to the media, they form a sticky floating white precipitate and, while we tried to recover the whole remaining fatty alcohol by adding organic solvent directly to the tube, some of it may still remain on the walls of the tube.

**Table 1:** Strains used in this study.

Name	ID	Genotype	Reference
*S. cerevisiae* reference	CEN.PK113-7D	*MATa*	Entian and Kötter, 2007
*S. cerevisiae* non-producer	ST6849	pex10Δ, hfd1Δ, MATa	This study
*S. cerevisiae* fatty alcohol producer	ST6989	*4xMalFAR*, pex10Δ, hfd1Δ, *MATa*	This study
*Y. lipolytica* reference	W29	MATa	Gaillardin et al., 1973
*Y. lipolytica* non-producer	ST6770	pex10Δ, fao1Δ, hfd1Δ, hfd4Δ, *LEU2*, *LYS11*, *URA3*, MATb, *ku70*Δ, *nugm-Htg2*, *ndh2i*, *lys11^−^*, *leu2^−^*, *ura3^−^*	This study
*Y. lipolytica* fatty alcohol producer	ST6987	*4xMalFAR*, pex10Δ, fao1Δ, hfd1Δ, hfd4Δ, *LEU2*, *LYS11*, *URA3*, MATb, *ku70*Δ, *nugm-Htg2*, *ndh2i*, *lys11^−^*, *leu2^−^*, *ura3^−^*	This study

### RNA Sequencing and Gene Expression Analysis

Growth phase cultures were sampled at OD 2–3 (∼6 generations), when the cultures were in the mid-exponential growth phase and the unlabeled biomass from inoculum was diluted to less than 3%. Nitrogen-depleted cultures were sampled when ca. 50% of glucose was consumed, which was after 12 h for *S. cerevisiae* and after 48 h for *Y. lipolytica*. The sample volume corresponding to 5 × 10^7^–10^8^ cells was added into 50-ml Falcon tube, filled with ice. The tubes were centrifuged at 4°C for 1 min, the liquid was discarded, and the pellet was snap frozen in liquid nitrogen and stored at −80°C until further processing. The cell lysis was carried out in 2 ml-screw cap tubes with 600-µl RLT buffer and 500-µl glass beads using a Precellys 24 at 6000 RPM for 4 × 25 s, with 60 s on ice in between. RNA was subsequently extracted using the RNeasy kit (Qiagen), according to the manufacturer’s instructions.

Library preparation was carried out using the TruSeq Stranded mRNA Library Prep Kit (Illumina), and the TruSeq RNA CD indexes (Illumina). Sequencing was carried out using a NextSeq 500 system (Illumina), with NextSeq Mid and High Output v2 Kits (150 cycles), as 75 bp paired-end reads. Index (i7 and i5) reads: 8 bp, flow cell loading: 1.08 pM, sequencing chemistry: 2-channel sequencing-by-synthesis (SBS) technology. PhiX was added at 2.5%. Sequencing facility: NGS lab at the Novo Nordisk Foundation Center for Biosustainability.

The RNA-seq data was processed using KBase (Arkin et al., 2018), and unless specified, default settings were used. Reads were trimmed using Trimmomatic v0.36 (post-tail crop length: 73, head crop length: 14) (Bolger et al., 2014). Read quality was assessed by FastQC. Reads were merged using Multiple ReadsLibs to One ReadsLib v1.0.1. Reads were aligned to the reference genome using HISAT2 v2.1.0 (Kim et al., 2015). The reference genomes used were modified versions of S288C and W29 (Clib89) for *S. cerevisiae* and *Y. lipolytica*, respectively; the modification consisted of the addition of the expressed *malFAR* genes. Alignment quality was assessed using Qualimap2 v2.2.1 (Okonechnikov et al., 2016). Alignments were assembled using StringTie v1.3.3b (not allowing for novel transcripts) (Pertea et al., 2015). The analysis was carried out separately using EdgeR v3.24.3 (Robinson et al., 2010) in R v3.5.1, using TMM normalization as well as false discovery rate (FDR) correction using the Benjamini–Hochberg method. EdgeR was used to calculate the effect of either nitrogen-depletion or fatty alcohol production in both species. Contrasts were set as “(A+B)/2 − (C+D)/2” where A and B belonged to the same condition (e.g., growth phase), and C and D belonged to the same condition (e.g., nitrogen depletion) An exception was made for the effect of fatty alcohol production in *Y. lipolytica*, where only genes differentially expressed between strains in nitrogen-depleted conditions were considered, due to a lack of difference between the strains during growth phase. Differentially expressed genes predicted by EdgeR were filtered for a *p*-value or 0.01, log2 CPM of 1, and log2-fold change of 2.

GO term enrichment analysis was carried out by the PANTHER v14.0–based online tool (Ashburner et al., 2000; Thomas et al., 2003; Mi et al., 2017; The Gene Ontology Consortium, 2017) available at www.geneontology.org. Enrichment analysis was carried out for biological process GO terms using Fisher’s exact test and Bonferroni correction. Results were filtered for a p-value less than 0.05 and fold enrichment greater than 2.5.

RNA-seq data has been uploaded to European Nucleotide Archive (PRJEB32352). A list of differentially expressed genes is attached as **Supplementary File S3**.

### ^13^C-Metabolic Flux Analysis

Cells were cultivated as previously described (*Cultivation*), with the exception that labeled glucose was used. One replicate was made with 20% U–^13^C glucose (99% purity, Euriso-Top GmbH, Saarbrücken, Germany) and 80% non-labeled glucose. Two replicates were made with 20% U–^13^C glucose and 80% 1–^13^C glucose (99% purity, Euriso-Top GmbH, Saarbrücken, Germany). The two isotopomers (1–^13^C glucose and U–^13^C glucose) are commonly used in ^13^Cmetabolic flux analysis as they provide a good flux resolution at a reasonable cost. Furthermore, the single replicate of 20% U^13^C glucose can be used to estimate the quality of the MS data. The labeling strategy was informed by previous publication (Zamboni et al., 2009). A cell amount of 0.3 mg CDW were harvested at OD 2 (∼6 generations) and washed with 1 ml cold NaCl (0.9%). The pellet was stored at −80°C until processing. The samples were resuspended in 150 μl HCl (6 M), transferred to a glass vial, and incubated at 105°C for 6 h to hydrolyze the cell pellet as previously described (Schmitz et al., 2017). Samples were dried until only a dark brown residue remained by heating the open vial at 80°C under a fume hood. The residue was resuspended in 30 μl acetonitrile. Samples were derivatized by the addition of MBDSTFA at a 1:1 ratio and incubated at 85°C for 1 h. Samples were analyzed by GC-MS according to a previously described protocol (Kildegaard et al., 2016). Raw GC-MS data was corrected using iMS2Flux v.7.2.1 (Poskar et al., 2012). Fluxes were calculated using parameter continuation in the INCA v1.7 software (Young, 2014). The model used was adapted from previous publication (Wasylenko and Stephanolous, 2015), see **Supplementary File S4** for the final model used, as well as for the full set of calculated fluxes.

### Intracellular Metabolome Analysis

Samples corresponding to approximately 0.25 mg CDW were taken for each replicate. For growth phase samples, samples were taken at mid-growth phase (OD 1–2, ∼6 generations). For the nitrogen-depletion samples, samples were taken at 12 or 48 h, respectively, for *S. cerevisiae* and *Y. lipolytica* strains. The sampling method used was adapted from a previous publication (McCloskey et al., 2015b), with the addition of an extra extraction step, in which the filter with the quenched biomass was transferred into a 50-ml Falcon tube containing 5 ml boiling ethanol, together with the 1 ml extraction solvent used for quenching (containing internal standard). The filter was incubated at 80°C in the boiling ethanol for 90 s, the tube was quickly vortexed, the filter was flipped, and the tube was incubated for another 90 s. The solution was aliquoted into 2-ml Eppendorf tubes and processed according to the previously described protocol. See **Supplementary File S5** for full details. The analysis was conducted according to previous publication (McCloskey et al., 2015a).

## Results

### Establishing Fatty Alcohol Production in *S. cerevisiae* and *Y. lipolytica*

**Figure 2:**
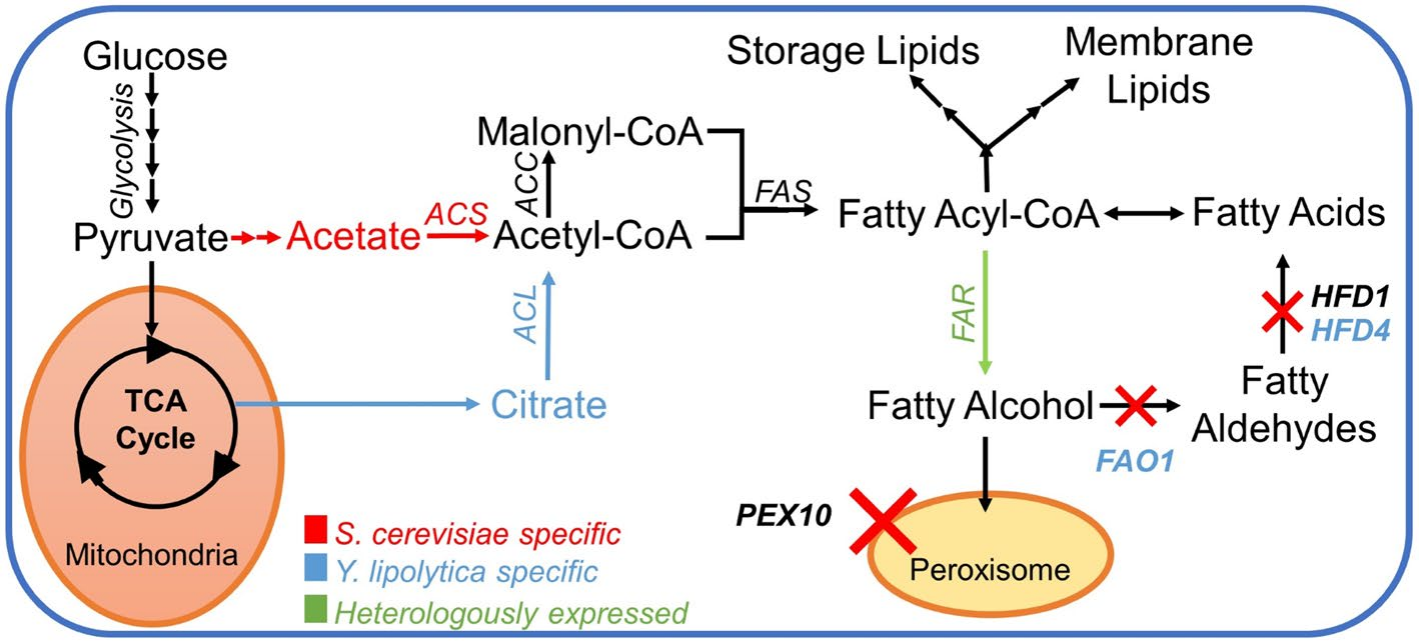
Pathway for fatty alcohol production; a simplified pathway for the metabolic conversion of glucose into fatty alcohols in *S. cerevisiae* and *Y. lipolytica*. Color-coded text and arrows: red, pathway in *Saccharomyces cerevisiae*; blue, pathway in *Yarrowia lipolytica*; green, heterologous reactions. The red X-symbol signifies corresponding gene knockouts. Abbreviations: *ACL*, ATP citrate lyase; *ACS*, acetyl–CoA synthase; *ACC*, acetylCoA carboxylase; *FAS*, fatty acid synthase complex; *FAR*, fatty acyl–CoA reductase; *PEX10*, peroxin 10; *FAO1*, fatty alcohol oxidase; *HFD1*, fatty aldehyde dehydrogenase 1 (*ALDH1*); *HFD4*, fatty aldehyde dehydrogenase 4 (*ALDH4*).

The first step towards creating fatty alcohol–producing yeast strains was to reduce the degradation of fatty alcohols as described in Borodina et al. (2018). In *S. cerevisiae*, we chose to delete the genes encoding peroxisomal biogenesis factor Pex10p and aldehyde dehydrogenase Hfd1p (**Figure 2**). The deletion of *PEX10* prevents the formation of peroxisomes, where β-oxidation of fatty acids occurs. The *HFD1* gene was shown in a previous study (Buijs et al., 2015) to be responsible for the degradation of fatty alcohols in *S. cerevisiae*. In *Y. lipolytica*, we deleted *PEX10* and two out of four aldehyde dehydrogenase-coding genes *HFD1* and *HFD4*, the ones that were previously reported to have the highest activity (Iwama et al., 2014). Additionally, we deleted the *FAO1* gene encoding a fatty alcohol oxidase in *Y. lipolytica* (**Figure 2**).

**Figure 3:**
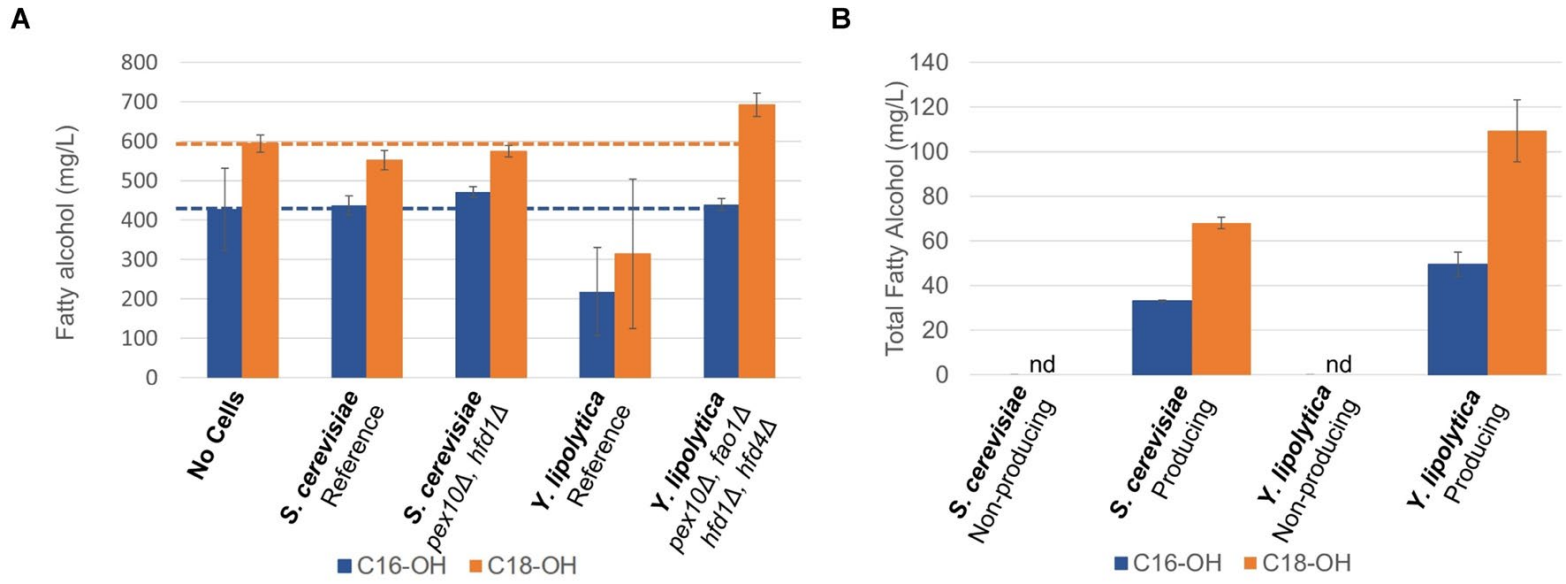
Fatty alcohol degradation and production **(A)** Fatty alcohol degradation was estimated by cultivating yeast strains in the presence of extracellular fatty alcohol mixture consisting of hexadecanol (C16-OH) and octadecanol (C18-OH) and quantifying the residual concentrations. **(B)** Production of fatty alcohols by engineered strains of *S. cerevisiae* and *Y. lipolytica* on minimal medium after 96 h, nd: not detected. Data shown are mean values ± standard deviations of biological triplicates.

In order to investigate if the chosen gene knockouts reduced the degradation of fatty alcohols, we cultivated the non-engineered and engineered strains in the medium supplemented with approximately 0.5 g/L each of hexadecanol and octadecanol for 96 h and analyzed the remaining fatty alcohol concentration (**Figure 3A**). There was no significant difference between the final concentrations of fatty alcohols between the cultures of *S. cerevisiae* strains and the control experiment without cell addition. As it has been shown previously that *S. cerevisiae* can degrade fatty alcohols (d’Espaux et al., 2017) that it produces, the lack of apparent degradation of extracellular fatty alcohols could be explained by poor uptake. As for *Y. lipolytica*, the reference strain degraded approximately half of the added hexadecanol and octadecanol, whereas the engineered strain (*pex10Δ, fao1Δ, hfd1Δ, hfd4Δ*) showed no fatty alcohol degradation, indicating that the knockouts had impaired the ability of the strain to degrade fatty alcohols, as intended.

In the next step, we integrated four copies of the *malFAR* fatty acyl–CoA reductase (*FAR*) gene into the yeast strains with reduced fatty alcohol degradation. The *FAR* genes were expressed from strong constitutive promoters, *TEF1* and *TDH3* (*GPD*) for *S. cerevisiae* and from *TEFintron* (Tai and Stephanopoulos, 2013) and *GPD* promoters for *Y. lipolytica*. The strong constitutive promoters were selected to ensure that fatty alcohol biosynthetic genes were expressed both in the growth phase and in the nitrogen-depletion phases. The fatty alcohol production was evaluated in small-scale shake flask cultivations in mineral media for 96 h (**Figure 3B**). The *S. cerevisiae* strain carrying the *FAR* produced 105 ± 3 mg/L of total fatty alcohols, whereas the *Y. lipolytica* strain produced 166 ± 20 mg/L of total fatty alcohols. These titers are similar to the shake flasks titers reported by previous study (Xu et al., 2016). Xu et al. subsequently reported 2.2 g/L in bioreactors using the same strains, the highest reported levels on minimal media.

Throughout this study, non-producing strains of *S. cerevisiae* (*pex10Δ, hfd1Δ*) and *Y. lipolytica* (*pex10Δ, fao1Δ, hfd1Δ, hfd4Δ*) were compared with fatty alcohol–producing strains of *S. cerevisiae* (4x *malFAR*, *pex10Δ, hfd1Δ*) and *Y. lipolytica* (4x *malFAR*, *pex10Δ, fao1Δ, hfd1Δ, hfd4Δ*), during both the exponential growth phase as well as during a nitrogen-depleted stationary phase (**Table 1**). Pre-cultures were grown on minimal media until the late exponential phase, and subsequently washed and resuspended in fresh minimal media for growth or in nitrogen-depletion media for stationary phase studies. This experimental set-up allowed for simplified parallel investigation of each phase individually, which could be made to occur in sequence as a result of nitrogen consumption in an industrial setting. The nitrogen-depleted stationary phase is of interest since it has previously been shown to increase the flux *via* fatty acyl–CoA to triacylglycerides and lipid accumulation (Pomraning et al., 2016).

**Figure 4:**
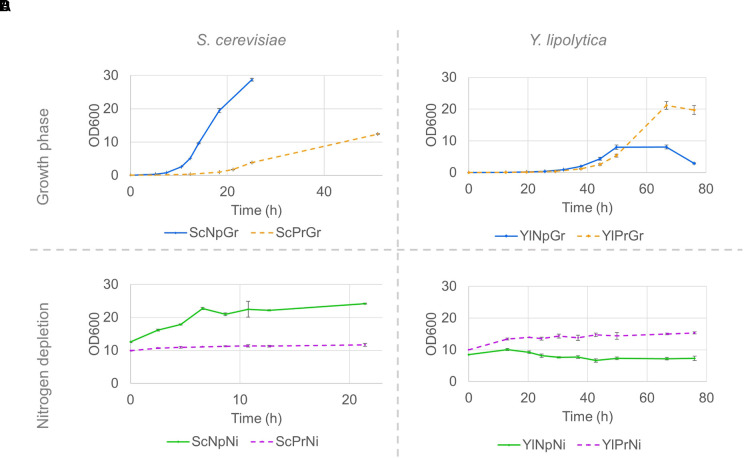
Strain growth in minimal media and nitrogen-depleted media; Growth curves of *S. cerevisiae* **(A**, **C)** and *Y. lipolytica* strains **(B**, **D)** on minimal media **(A**, **B)** and nitrogen-depletion media **(C**, **D)**. Pre-cultures were grown on minimal media until the late exponential phase, and subsequently washed and resuspended in either fresh minimal media or in nitrogen-depletion media. Data shown are mean values ± standard deviations of biological triplicates. Sc, ***S*** *accharomyces* ***c*** *erevisiae*; Yl, ***Y*** *arrowia* ***l*** *ipolytica*, Pr, fatty alcohol **pr**oducing; Np, **n**on-**p**roducing; Gr, **gr**owth phase; Ni, **ni**trogen-depleted stationary phase.

The fatty alcohol–producing strain of *S. cerevisiae* had a 50% lower maximum specific growth rate, µ_max_, and reached a lower final OD, ∼40% of the final OD reached by the parental strain not expressing *FAR* genes (**Figure 4A** and **Table 2**). Although no time series data is available beyond the 50 h, we made some separate experiments under the same conditions, and no further increase in OD was observed between 50 and 96 h for the producing strain. In contrast, the µ_max_ of *Y. lipolytica* was not affected by expression of fatty alcohol reductase genes, and the strain with fatty alcohol production grew to a 2.6-fold higher final OD (**Figure 4B** and **Table 2**). When the strains were transferred to a medium without nitrogen and cultivated for 21 h, the OD of the non-producing *S. cerevisiae* strain increased 1.9-fold (**Figure 4C**). The OD of the non-producing *Y. lipolytica* strain decreased slightly, indicating some negative effect of the introduced gene deletions (**Figure 4D**).

**Table 2:** Uptake-, secretion-, and growth rate. Sc, ***S*** *accharomyces* ***c*** *erevisiae*; Yl, ***Y*** *arrowia* ***l*** *ipolytica*, Pr, fatty alcohol **pr**oducing; Np, **n**on-**p**roducing; Gr, **gr**owth phase; Ni, **ni**trogen-depleted stationary phase; nd, not determined. Data shown are mean values ± standard deviations of biological triplicates.

	Glucose	Ethanol	Acetate	Glycerol	Succinate	Malate	Isocitrate
	mmol g^−1^ h^−1^	mmol g^−1^ h^−1^	mmol g^−1^ h^−1^	mmol g^−1^ h^−1^	µmol g^−1^ h^−1^	µmol g^−1^ h^−1^	µmol g^−1^ h^−1^
**ScNpGr**	15.7 ± 0.29	22. ± 0.3	1.41 ± 0.1	2.81 ± 0.0	nd	nd	nd
**ScPrGr**	15.4 ± 0.26	20.6 ± 0.4	2.3 ± 0.3	3.89 ± 0.3	nd	nd	nd
**ScNpNi**	1.17 ± 0.12	0.9 ± 0.5	0.2 ± 0.0	0.0 ± 0.0	nd	nd	nd
**ScPrNi**	1.61 ± 0.10	2.4 ± 0.9	0.1 ± 0.0	0.3 ± 0.0	nd	nd	nd
**YlNpGr**	1.52 ± 0.19	nd	nd	nd	1.2 ± 0.4	8.3 ± 1.9	1.1 ± 0.1
**YlPrGr**	2.04 ± 0.20	nd	nd	nd	0.8 ± 0.0	5.5 ± 0.2	0.4 ± 0.0
**YlNpNi**	0.35 ± 0.01	nd	nd	nd	5.0 ± 0.7	31.3 ± 3.8	6.6 ± 1.2
**YlPrNi**	0.22 ± 0.03	nd	nd	nd	1.8 ± 0.3	9.1 ± 0.6	0.2 ± 0.5
	**Citrate**	**α-Ketoglutarate**	**Fumarate**	**Pyruvate**	**Fatty alcohols**	**Growth rate**	
	µmol g^−1^ h^−1^	µmol g^−1^ h^−1^	µmol g^−1^ h^−1^	µmol g^−1^ h^−1^	µmol g^−1^ h^−1^	h^−1^	
**ScNpGr**	nd	nd	nd	nd	nd	0.33 ± 0.00	
**ScPrGr**	nd	nd	nd	nd	nd	0.17 ± 0.00	
**ScNpNi**	nd	nd	nd	nd	nd	nd	
**ScPrNi**	nd	nd	nd	nd	1.9 ± 0.7	nd	
**YlNpGr**	1.1 ± 0.8	30.2 ± 3.9	1.7 ± 0.4	79.7 ± 8.9	nd	0.13 ± 0.00	
**YlPrGr**	0.6 ± 0.1	11.9 ± 0.9	1.0 ± 0.1	33.2 ± 5.0	nd	0.12 ± 0.00	
**YlNpNi**	54.9 ± 3.2	39.1 ± 13.4	7.2 ± 1.4	102.7 ± 5.4	nd	nd	
**YlPrNi**	12.6 ± 1.2	19.9 ± 2.2	1.3 ± 0.1	29.9 ± 2.7	6.0 ± 0.9	nd	

Glucose uptake rates varied greatly between the hosts and conditions. *S. cerevisiae* exhibited a ∼13-fold and ∼5-fold higher glucose uptake rate in growth phase and nitrogen-depleted stationary phase, respectively, compared to *Y. lipolytica*. Furthermore, the uptake rate was ∼17-fold higher and ∼6-fold higher in the growth phase than in the nitrogen-depletion phase in *S. cerevisiae* and *Y. lipolytica*, respectively (**Table 2**).

As for by-products, *S. cerevisiae* primarily secreted ethanol, as well as some acetate and glycerol. There was no major change in the by-product secretion of the producing and non-producing strains of *S. cerevisiae*. *Y. lipolytica* secreted several TCA cycle–associated organic acids, primarily pyruvate, α-ketoglutarate, citrate, and malate. The fatty alcohol–producing strain of *Y. lipolytica* exhibited a 2–3-fold lower by-product secretion rates for all the measured metabolites, during both exponential growth and nitrogen-depleted stationary phase, plausibly due to a redirection of the flux toward fatty alcohols. The relative secretion rate (relative to glucose uptake rate) of isocitrate in the producing *Y. lipolytica* strain was reduced by ∼20-fold in the nitrogen-depleted condition. The reduced isocitrate secretion rate could potentially be explained by an increased transport of mitochondrial citrate to the cytoplasm, followed by conversion of citrate to acetyl–CoA (catalyzed by ATP-citrate lyase), which in turn would be used for fatty alcohol production. In response to nitrogen-depletion, *Y. lipolytica* exhibited a 5–25-fold relative increase in secretion rate of all by-products analyzed, except for citrate, which was secreted with a ∼200-fold increased relative rate. During nitrogen-depletion, *Y. lipolytica* secreted ∼50% and ∼25% of the total carbon consumed, in the form of organic acid by-products for the non-producing and producing strain, respectively. Additionally, the fatty alcohol–producing strain produced fatty alcohols corresponding to ∼8% of the carbon consumed. Both *S. cerevisiae* and *Y. lipolytica* showed detectable fatty alcohol production only in nitrogen-depleted conditions (**Table 2**).

### ^13^C-Metabolic Flux Analysis

In order to get insight into the strains’ response to fatty alcohol production on a metabolic level, ^13^C-fluxomics and targeted metabolomics analyses were conducted. The flux analysis determines the fluxes (conversion rates) between metabolites. Fluxes reveal the flow of carbon through the cell and the distribution of carbon flux between alternative pathways. For ^13^C-flux analysis, the cells were cultivated on a mix of labeled and unlabeled glucose. The incorporation of labeled carbons (^13^C) into proteinogenic amino acids was measured by GC-MS. The ^13^C-flux analysis method requires a metabolic steady state and, if based on measurements of proteinogenic amino acids, growing cells. Therefore, the fluxes were only estimated for the exponential growth phase, which can be considered to represent a quasi-steady-state condition.

**Figure 5:**
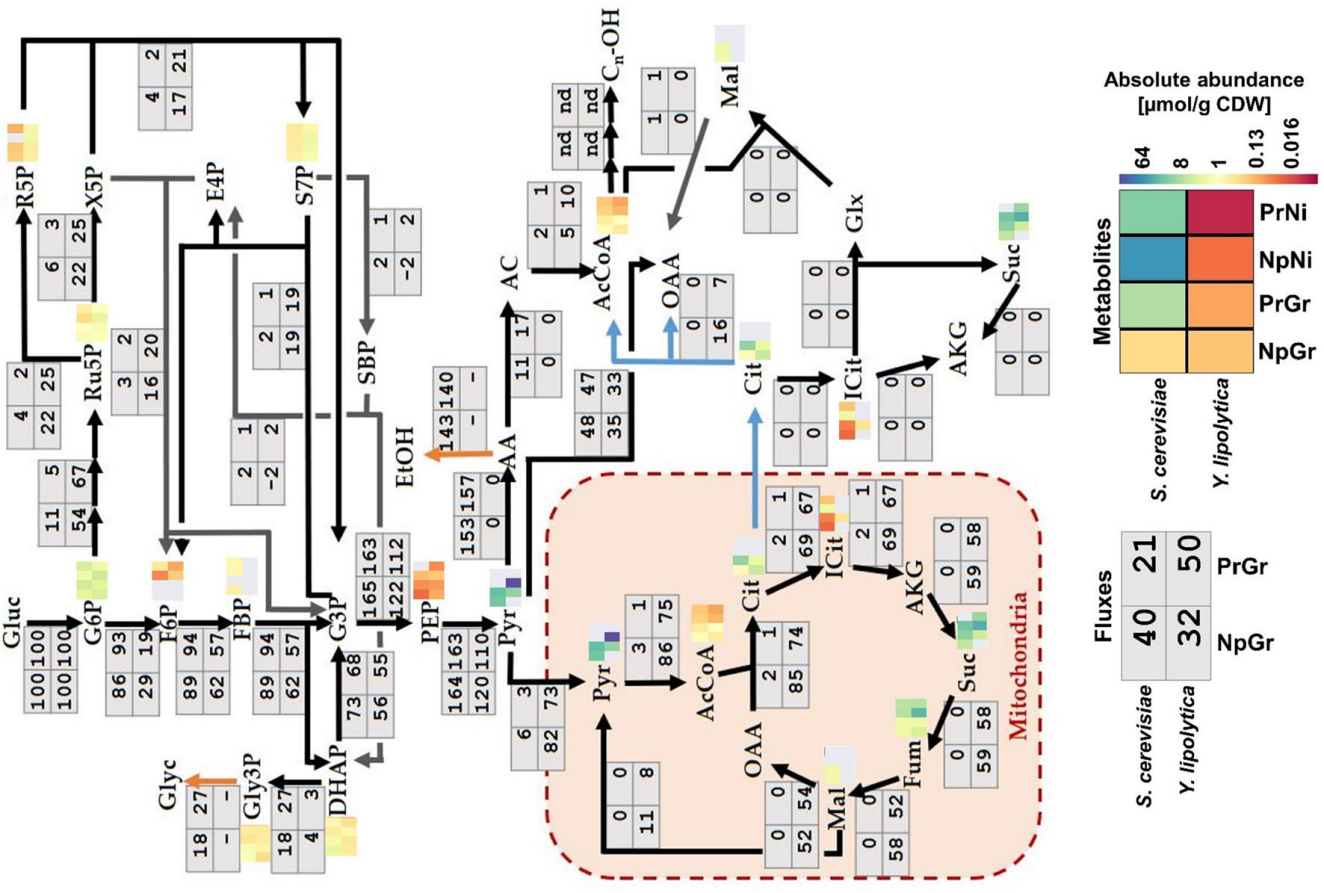
Fluxes and metabolite levels in central carbon metabolism. Fluxes (gray boxes) are relative values, defined as the percentage of glucose uptake rate. Negative values indicate that the reaction is taking place in the opposite direction to what is indicated by the arrow. Metabolites (heatmaps) correspond to absolute abundances in µmol/g CDW; gray means data missing (below the detection limit, saturated signal, or too high background in control sample). Sc: ***S*** *accharomyces* ***c*** *erevisiae*, Yl: ***Y*** *arrowia* ***l*** *ipolytica*, Pr: fatty alcohol **pr**oducing, Np: **n**on-**p**roducing, Gr: **gr**owth phase, Ni: **ni**trogen-depleted stationary phase.

The central carbon flux distributions in *S. cerevisiae* and *Y. lipolytica* were very different (**Figure 5**). The *S. cerevisiae* strains processed approximately 90% of the carbon from glucose through glycolysis, whereas only around 10% of the carbon went into the pentose phosphate pathway. The cells channeled ∼50% of the carbon from glucose to ethanol, primarily relying on the energy generated in the fermentation process. *Y. lipolytica* is a non-fermenting yeast, so the energy is generated by oxidative phosphorylation in the mitochondria. Besides the higher TCA cycle flux associated with this respiratory lifestyle, *Y. lipolytica* differed from *S. cerevisiae* by diverting nearly half of the internalized glucose into the pentose phosphate pathway generating substantial amounts of NADPH. NADPH is a key redox co-factor for fatty acid and fatty alcohol biosynthesis. Additionally, *Y. lipolytica* had a ∼5-fold higher relative flux (relative to glucose uptake) towards cytosolic acetyl–CoA than *S. cerevisiae*. Acetyl–CoA is the precursor for fatty acyl-CoA, which in turn is the precursor for fatty alcohols, and other fatty acid–derived compounds. It is worth noting that *S. cerevisiae* and *Y. lipolytica* utilize different pathways for the synthesis of acetyl–CoA from glucose (**Figure 2**). In *S. cerevisiae*, most of cytosolic acetyl–CoA is produced by the action of pyruvate decarboxylase, aldehyde dehydrogenase, and acetyl–CoA synthase. The NADP-dependent *ALD6* has previously been shown to contribute ∼40% of the NADPH generated in *S. cerevisiae*, with the remaining 60% being generated *via* the pentose phosphate pathway (Blank et al., 2005). In contrast, *Y. lipolytica* uses ATP-citrate lyase (*ACL*) to make acetyl–CoA from citrate, which is exported from mitochondria with a simultaneous import of malate. The by-product of the ACL reaction is oxaloacetate, which is converted into malate. In *Y. lipolytica*, the NADPH is primarily generated through the pentose phosphate pathway (Wasylenko et al. 2015).

### Metabolomics

Metabolomics analysis determines the intracellular metabolite concentrations. Metabolite pools can help to reveal limitations in precursor availability, as well as potentially limiting steps in the production pathway. Samples for metabolome analysis were taken during growth or nitrogen-depletion in producing and non-producing strains of both *S. cerevisiae* and *Y. lipolytica* and were rapidly filtered and quenched.

The metabolomics data revealed a decreased abundance of acetyl–CoA during nitrogen-depletion in both species, ∼3- and ∼6.5-fold lower in *S. cerevisiae* and *Y. lipolytica*, respectively (**Figure 5**). This might indicate that fatty alcohol–producing strains encounter a limited precursor supply under these conditions. *Y. lipolytica* had increased levels of intermediates of the pentose phosphate pathway, which is consistent with the high flux through this pathway. Ribose 5-phosphate (r5p), ribulose 5-phosphate (ru5p), and sedoheptulose 7-phosphate (s7p) had a ∼3-fold higher abundance in *Y. lipolytica* compared to *S. cerevisiae* strain. Given that this difference was observed with both producing and non-producing strains, it appears to be an inherent feature of *Y. lipolytica* metabolism.

### Transcriptomics

**Figure 6:**
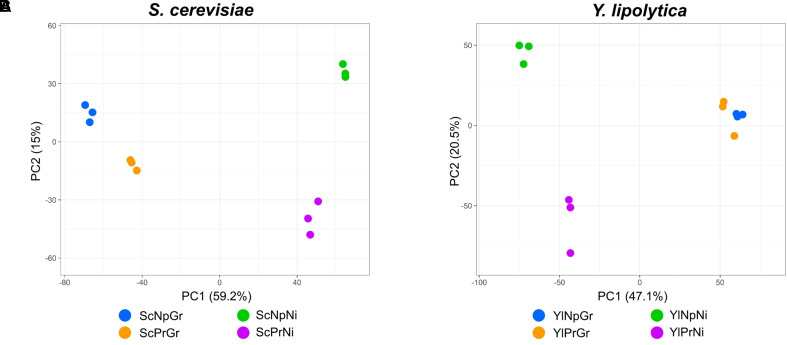
Principal component analysis of transcriptomic data; PCA scatterplot showing *S. cerevisiae* RNA-seq samples projected onto the first two principal components of the data **(A)**. PCA scatterplot showing *Y. lipolytica* RNA-seq samples projected onto the first two principal components of the data **(B)**. Sc, ***S*** *accharomyces* ***c*** *erevisiae*; Yl, ***Y*** *arrowia* ***l*** *ipolytica;* Pr, fatty alcohol **pr**oducing; Np, **n**on-**p**roducing; G, **gr**owth phase; Ni, **ni**trogen-depleted stationary phase.

The gene expression profiles of fatty alcohol producing and non-producing strains were analyzed during growth and nitrogen-depletion *via* RNA sequencing. Principal component analysis (PCA) plots of the data revealed four separate sample groups in *S. cerevisiae* and three separate groups in *Y. lipolytica* (**Figure 6**). In *S. cerevisiae*, these groups represented the four different conditions (fatty alcohol–producing strain in growth and nitrogen-depletion, and non-producing strain in growth and nitrogen-depletion), indicating clear differences between all analyzed strains/conditions. In contrast, for *Y. lipolytica*, the three groups revealed that the producing and non-producing strains in the nitrogen-depletion phase were clearly different from each other and from the strains in the growth phase. But producing and non-producing strains in the growth phase were similar. Furthermore, direct comparison of the differentially expressed genes between the two conditions revealed only nine functionally annotated genes, out of which only four were also differentially expressed between the same two strains in nitrogen-depleted conditions. Considering this similarity, only *Y. lipolytica* strains subjected to the nitrogen-depleted conditions were used to identify the differentially expressed genes in response to fatty alcohol production in *Y. lipolytica*. In both species, PC1 separates growth phase from nitrogen-depleted stationary phase and explains 59% and 47% of the difference in *S. cerevisiae* and *Y. lipolytica*, respectively.

In response to nitrogen-depletion (nitrogen-depleted stationary phase *vs*. growth phase), *S. cerevisiae* differentially expressed 716 genes, 401 of which were upregulated in nitrogen-depleted stationary phase, and 315 were downregulated (**Table 3**). Out of the 331 characterized upregulated genes, the most enriched GO terms were those related to the TCA cycle, carbohydrate metabolism, as well as various metabolic processes. These points toward a shift in the metabolic profile, switching from the rapid but wasteful Crabtree overflow metabolism to a more energy conservative strategy utilizing the mitochondria, and storing excess carbon as glycogen and trehalose. Furthermore, the GO categories “response to oxidative stress” and “response to toxic substance” were also enriched. The 286 characterized downregulated genes were enriched for ribosome-/translation-related processes as well as RNA metabolic processes. The enrichment analysis of the downregulated genes indicated a slowdown of the central cellular processes associated with adaptation to the stationary phase, i.e., quiescence (Coller, 2011), induced by the nitrogen depletion. The response is similar to what has been seen in previous studies (Boer et al., 2003), and even though it is not revealed in the enrichment analysis, individual inspection of differentially expressed genes showed that the strains underwent transcriptional changes associated with the release of nitrogen catabolite repression (Daugherty et al., 1993; ter Schure et al., 2000) (**Table S3**). Nitrogen depletion triggered the upregulation of nitrogen transporters such as *GAP1* (general amino acid permease, YKR039W) and *PUT4* (proline permease, YOR348C), as well as the upregulation of enzymes involved in nitrogen metabolism such as *DUR1,2* (urea amidolyase, YBR208C) and *DAL1* (allantoinase, YIR027C).

**Table 3:** GO term enrichment in differentially expressed genes. Go terms enriched more than 2.5-fold. Indented GO terms in brackets are a sub-group of the preceeding broader higher-level GO term. Differentially expressed genes described as condition 1 *vs*. condition 2, where upregulated signifies that condition 1 has a higher transcript abundance, and downregulated signifies that condition 2 has a higher transcript abundance. Uncharacterized genes consist of unclassified or unknown genes.

*S. cerevisiae:* nitrogen depletion *vs*. growth				
**Upregulated genes**				
Total: 401 genes, uncharacterized: 70 genes				
**GO biological process complete**	**Gene count (331)**	**Expected**	**Fold enrichment**	**P-value**
Glyoxylate metabolic process (GO:0046487)	6	0.6	**11**	9.60E-05
Oligopeptide transmembrane transport (GO:0035672)	5	0.5	**10.2**	5.00E-04
Glutamate metabolic process (GO:0006536)	6	0.9	**6.5**	8.90E-04
Antibiotic metabolic process (GO:0016999)	18	3.4	**5.3**	8.70E-08
[Tricarboxylic acid cycle (GO:0006099)]	11	1.6	**6.7**	4.80E-06
*‘De novo*’ protein folding (GO:0006458)	7	1.3	**5.3**	8.50E-04
Ammonium transport (GO:0015696)	6	1.2	**5.2**	2.20E-03
Carbohydrate biosynthetic process (GO:0016051)	17	3.5	**4.9**	4.80E-07
Monosaccharide metabolic process (GO:0005996)	16	3.7	**4.4**	4.10E-06
Carbohydrate catabolic process (GO:0016052)	20	4.7	**4.3**	3.10E-07
Response to toxic substance (GO:0009636)	15	3.8	**3.9**	2.50E-05
Monocarboxylic acid metabolic process (GO:0032787)	35	3.8	**3.8**	1.50E-10
Carbohydrate metabolic process (GO:0005975)	50	13.2	**3.8**	1.70E-14
Small molecule catabolic process (GO:0044282)	30	8	**3.8**	5.00E-09
Response to oxidative stress (GO:0006979)	25	6.6	**3.8**	8.00E-08
Cellular glucan metabolic process (GO:0006073)	11	3	**3.7**	4.50E-04
Oxidation-reduction process (GO:0055114)	75	24.1	**3.1**	2.30E-17
[Carboxylic acid catabolic process (GO:0046395)]	15	4.8	**3.2**	2.20E-04
[Fatty acid beta-oxidation (GO:0006635)]	7	0.7	**10.7**	2.80E-05
Response to heat (GO:0009408)	15	4.9	**3.1**	2.70E-04
Drug metabolic process (GO:0017144)	43	15.3	**2.8**	6.10E-09
Cellular response to external stimulus (GO:0071496)	18	6.7	**2.7**	3.30E-04
Nucleobase-containing small molecule metabolic process (GO:0055086)	41	15.7	**2.6**	9.80E-08
[Nucleotide metabolic process (GO:0009117)]	34	13.6	**2.5**	3.00E-06
Cofactor metabolic process (GO:0051186)	37	14	**2.6**	4.20E-07
Coenzyme metabolic process (GO:0006732)	26	10.3	**2.5**	5.00E-05
[Glycolytic process (GO:0006096)]	7	1.4	**4.9**	1.30E-03
**Downregulated genes**			****	
Total: 315 genes, uncharacterized: 29 genes				
**GO biological process complete**	**Gene count (293)**	**Expected**	**Fold enrichment**	**P-value**
S-adenosylmethionine metabolic process (GO:0046500)	5	0.3	**14.8**	1.20E-04
Regulation of establishment or maintenance of cell polarity (GO:0032878)	4	0.3	**11.8**	1.10E-03
Nucleobase biosynthetic process (GO:0046112)	7	1.1	**6.6**	2.70E-04
DNA integration (GO:0015074)	14	2.4	**5.8**	8.80E-07
rRNA-containing ribonucleoprotein complex export from nucleus (GO:0071428)	14	3.1	**4.6**	8.90E-06
Ribosomal small subunit biogenesis (GO:0042274)	28	7	**4**	3.60E-09
DNA biosynthetic process (GO:0071897)	16	4.7	**3.4**	4.90E-05
Amide biosynthetic process (GO:0043604)	65	20.3	**3.2**	6.40E-16
[Translation (GO:0006412)]	61	17.7	**3.4**	3.10E-16
Transposition, RNA-mediated (GO:0032197)	14	4.4	**3.2**	3.00E-04
Ribosomal large subunit biogenesis (GO:0042273)	18	5.9	**3.1**	6.90E-05
Cellular amino acid metabolic process (GO:0006520)	37	12.3	**3**	1.10E-08
RNA phosphodiester bond hydrolysis (GO:0090501)	27	9.1	**3**	1.70E-06
Cellular amide metabolic process (GO:0043603)	69	23.6	**2.9**	4.30E-15
Nucleoside phosphate biosynthetic process (GO:1901293)	19	7.4	**2.6**	3.10E-04
*S. cerevisiae*: producing *vs*. non-producing				
**Upregulated genes**				
Total: 21 genes, uncharacterized: four genes				
**GO biological process complete**	**Gene count (18)**	**Expected**	**Fold enrichment**	**P-value**
Cell wall organization or biogenesis (GO:0071554)	9	0.8	**11.0**	7.2E-05
**Downregulated genes**	****	****	****	****
Total: three genes, uncharacterized: three genes				
**GO biological process complete**	**Gene count (0)**	**Expected**	**Fold enrichment**	**P-value**
None	–	–	–	–
*Y. lipolytica*: nitrogen depletion *vs*. growth				
**Upregulated genes**	****	****	****	****
Total: 500 genes, uncharacterized: 355 genes				
**GO biological process complete**	**Gene count (315)**	**Expected**	**Fold enrichment**	**P-value**
Transmembrane transport (GO:0055085)	70	20.6	**3.4**	2.8E-18
[Ammonium transmembrane transport (GO:0072488)]	9	0.6	**15.4**	1.7E-07
[Carboxylic acid transmembrane transport (GO:1905039)]	11	2.3	**4.9**	5.1E-05
[Amino acid transmembrane transport (GO:0003333)]	11	1.6	**6.8**	3.6E-06
**Downregulated genes**	****	****	****	****
Total: 131 genes, uncharacterized: 54 genes				
**GO biological process complete**	**Gene count (121)**	**Expected**	**Fold enrichment**	**P-value**
Sulfur compound metabolic process (GO:0006790)	13	1.8	**7.3**	6.9E-08
*Y. lipolytica* producing *vs*. non-producing				
**Upregulated genes**	****	****	****	****
Total: 60 genes, uncharacterized: 21 genes				
**GO biological process complete**	**Gene count (43)**	**Expected**	**Fold enrichment**	**P-value**
Antibiotic catabolic process (GO:0017001)	6	0.1	**42.8**	1.57E-08
[Formate catabolic process (GO:0042183)]	4	0.1	**60.0**	1.59E-06
**Downregulated genes**	****	****	****	****
Total: 155 genes, uncharacterized: 79 genes				
**GO biological process complete**	**Gene count (120)**	**Expected**	**Fold enrichment**	**P-value**
Transport (GO:0006810)	51	16.5	**3.1**	2.2E-11
[Nitrogen compound transport (GO:0071705)]	26	7.7	**3.4**	6.8E-05
[Carboxylic acid transmembrane transport (GO:1905039)]	11	1.3	**8.7**	1.7E-04
[Amino acid transmembrane transport (GO:0003333)]	10	0.8	**12.1**	4.3E-05

The *Y. lipolytica* strains differentially expressed 631 genes in response to nitrogen depletion, out of which 500 were upregulated in nitrogen-depleted stationary phase, and 131 were downregulated. However, of the 500 upregulated genes 355 (71%) were uncharacterized. Of the 145 characterized upregulated genes, GO terms relating to transporters were the only terms significantly enriched. A large part of these belonged to transporters for nitrogen-containing compounds such as ammonium, amino acids, oligopeptides, and urea. The upregulation of these transporters is likely the result of the alleviation of nitrogen catabolite repression imposed in the presence of ammonia, which is a logical biological response as the cell needs to find alternative nitrogen sources. Furthermore, considering the large increase in carboxylic acid secretion rate in response to nitrogen depletion (**Table 2**), carboxylic acid transporters were of particular interest. However, due to genes being associated with multiple GO terms, all the 11 carboxylic acid transporters predicted to be enriched were also annotated as amino acid transporters. Hence, the enrichment analysis didn’t predict any known transporters of the TCA-associated organic acids. Of the 77 characterized down-regulated genes, only a single GO term, “sulfur compound metabolic process,” was enriched. This does not appear to be related to sulfur-containing amino acids, but rather to the metabolism of other various sulfur-containing compounds possibly due to the unintended consequence of lowering (but not depleting) the extracellular sulfate concentration when depleting the media of nitrogen, which is added to the media in the form of ammonium sulfate.

In response to fatty alcohol production (producing strain *vs*. non-producing strain), the *S. cerevisiae* strain differentially expressed 24 genes, 21 of which were upregulated in the producing strain (**Table 3**). GO term enrichment analysis revealed that 9 out of the 17 characterized upregulated genes were associated with “cell wall organization or biogenesis.” A previous meta-study (Arroyo et al., 2009) compared the transcriptional response to three different compounds (zymolase, congo red, and pneumocandins) triggering a cell wall stress response. Lowering the cutoff threshold of the differential gene expression from a 4-fold to a 2-fold increase [same as used by the cell wall stress studies (Lagorce et al., 2003; Boorsma et al., 2004; García et al., 2004; Rodríguez-Peña et al., 2005) in the meta-analysis] revealed a significant overlap. Out of the 18 genes upregulated in all three cell wall stress conditions, 16 were found to be upregulated in response to *FAR* expression, indicating that the *S. cerevisiae* fatty alcohol–producing strain is experiencing cell wall stress. No other GO terms were enriched. Three genes were downregulated, all of which were uncharacterized.

The *Y. lipolytica* strains differentially expressed 215 genes in response to fatty alcohol production, out of which 60 genes were upregulated, and 155 were downregulated in the producing strain. GO term enrichment analysis singled out antibiotic catabolic process (especially formate catabolism) as a key factor among the 39 characterized genes upregulated during fatty alcohol production. Three of the 10 most upregulated characterized genes were formate dehydrogenases (YALI0_B22506g, YALI0_B19976g, and YALI0_E14256g) with relatively high RNA abundance (CPM) and very low p-values (**Supplementary Table S3**), which further supports that the formate dehydrogenase upregulation is biologically significant. Formate dehydrogenases catalyze the reversible reaction between formate and carbon dioxide (formate + NAD^+^ ⇌ CO_2_ + NADH + H^+^). Upregulation of formate dehydrogenases was also found to correlate with lipid accumulation in a recent study (Zhang et al., 2019), but the biological significance of the upregulation remains unclear. Genes annotated with the broad GO term “transporters” were significantly enriched among the 76 downregulated characterized genes. Among the enriched transporters, the carboxylic acid transmembrane transporters were again of particular interest as downregulation of those genes could explain the decrease of byproduct secretion rate observed in response to fatty alcohol production (**Table 2**). However, due to genes being associated with multiple GO terms, out of the 11 transporters associated with carboxylic acids, 10 of them were amino acid transporters. The remaining transporter, YALI0_B19470g, was homologous to the *S. cerevisiae* transporter *JEN1*, annotated as a monocarboxylic acid transporter.

## Discussion

^13^C-flux analysis revealed fundamentally different metabolic profiles of the two yeast species *S. cerevisiae* and *Y. lipolytica* (**Figure 5**), which is in line with previous findings (Christen and Sauer, 2011). This has implications for engineering strategies. Given that fatty alcohols have a much higher energy content than glucose, energy efficiency and abundant reducing power (NADPH) will likely be key factors for high-performing cell factories. For *S. cerevisiae*, this means that removal of ethanol production and increased NADPH generation are necessary. Ethanol production is a major drain of carbon atoms and is energetically inefficient. As for NADPH generation, this could, for example, be achieved by re-routing more carbon through the pentose phosphate pathway; another solution might be to replace NAD-dependent glycolytic enzymes with NADP-dependent ones (Kildegaard et al., 2016). It is likely that fatty alcohol production in *Y. lipolytica* would also benefit from increased NADPH supply. An increased proportion of carbon going through the pentose phosphate pathway has previously been shown to improve lipid accumulation (Wasylenko et al., 2015). Efficient NADPH generation has also been achieved by expressing the NADP-dependent glycerol-3-phosphate dehydrogenase (*caGAPC*), with either an NADH kinase (*ylYEF1*) or a cytosolic NADP-dependent malic enzyme (*mcMCE2*) (Qiao et al., 2017).

Transcriptomic analysis comparing producing and non-producing strains of *S. cerevisiae* revealed a cell wall stress response. The cell wall stress response was indicated by both the GO term enrichment analysis (**Table 3**) as well as comparisons with previous studies (Arroyo et al., 2009). The cell wall–related upregulated genes have various functions. Crh1p and Crh2p are involved in cross-linking between the (1,6)-β-glucan and chitin (Cabib et al., 2007). Slt2p is the MAPK (mitogen-activated protein kinase) responsible for triggering the cell wall stress response. Cwp1p, Pir2p, Pir3p, Ncw2p, Cis3p, Sed1p, Pst1p, and Ccw14p among others are covalently attached structural components of the cell wall. The reason for the triggering is unclear, but could conceivably be due to some kind of disturbance of the cell envelope caused by the produced fatty alcohols. It is interesting that no such stress response appears to be triggered in *Y. lipolytica*. The reasons for this difference are undetermined, but one hypothesis is that *Y. lipolytica*’s lipid accumulating abilities allow it to incorporate the produced fatty alcohols into lipid bodies, reducing disturbance to the cell envelope such as the one observed in *S. cerevisiae*. In terms of design considerations, avoiding the toxic effects of fatty alcohols will likely be a part of any beneficial design. The cell wall stress response seen in *S. cerevisiae* might prove difficult to overcome by rational design; it is, however, possible that adaptive laboratory evolution selecting for increased growth in the presence of fatty alcohols could alleviate the problem.

In *Y. lipolytica*, toxic effects were instead revealed in the non-producing strain in the form of a growth defect (lower growth rate and final OD), which to some degree seems to be alleviated by the expression by the *FAR* (**Figures 4B, D**). The reasons for this phenomenon are unclear. However, we hypothesize that the growth defect might be due to the accumulation of medium chain fatty aldehydes due to a reaction between unsaturated long chain fatty acids and free radicals and/or molecular oxygen in *hfd*-negative cells as described in a previous study (Xu et al., 2017). The generation of free radicals might be worsened by the *PEX10* knockout, which disrupts the peroxisome biogenesis and leads to oxidative stress (Van der Leij, 1992). Given that *malFAR* likely acts upon fatty aldehydes as a transient intermediate (**Figure 1**), it is possible that *malFAR* is also able to act upon medium chain fatty aldehydes, converting them into medium chain fatty alcohols, which possibly have a reduced toxic effect. At this point, this is mere speculation, and further experiments are needed to validate the hypothesis. Given the relocalization of peroxisomal matrix proteins to the cytoplasm as a result of the *PEX10* knockout, it might prove a better approach to leave the peroxisome intact and all its enzymes enclosed. An alternative approach to *PEX10* deletion is to prevent β-oxidation by knocking out the *POX* enzymes responsible for the first step in the β-oxidation cycle.

Nitrogen depletion appears to have some major drawbacks, such as reduced cellular and metabolic activities (**Tables 2** and **3**). This can be due to quiescence, which is a beneficial adaptation in a natural environment (Coller, 2011). Several studies on the cellular response to nitrogen limitation were made (Morin et al., 2011; Kerkhoven et al., 2016; Pomraning et al., 2016); though more research is needed to apply this knowledge to improve the cellular performance under the nitrogen limitation.

Although the metabolomics data doesn’t directly reveal the limiting steps of fatty alcohol synthesis, it may help narrow down the possible options by the assumption that the limiting steps occur in metabolic pathways where data is lacking. The combination of the depleted acetyl–CoA pool (**Figure 5**), the low glucose uptake rate, and high organic acid secretion rate (**Table 2**) indicate that there are one or more limiting steps occurring upstream of acetyl–CoA with the glucose uptake and glucose phosphorylation and conversion to F6P/FBP as possible candidates. In the case of *S. cerevisiae*, the pyruvate/acetaldehyde/acetate conversion is a potential target. The secretion profile (**Table 2**) of *Y. lipolytica* indicates that there are limiting steps following both pyruvate (pyruvate translocase and/or pyruvate dehydrogenase complex) and citrate (ATP-citrate lyase), or a possible competition with the plasma membrane-bound pyruvate and citrate transporters.

Furthermore, in *Y. lipolytica*, nitrogen depletion results in a significant increase in organic acid secretion, with the non-producing strain secreting ∼50% of the total carbon consumed in the form of organic acids. Knocking out organic acid transporters might help to limit this carbon loss. Based on the correlation of changing secretion rates with differential gene expression, and excluding amino acid transporters, a single carboxylic acid transporter (YALI0_B19470g) could be predicted and might be a contributor to the organic acid secretion. However, there are likely multiple other carboxylic transporters being upregulated among the currently unknown genes. A recent study (Zhang et al., 2019) indicates that lowering the pH of the media may shift *Y. lipolytica* from citrate secretion to lipid accumulation. Furthermore, the secretion of organic acids could also be reduced by expressing downstream enzymes using cytosolic organic acids as substrates, such as ATP-citrate lyase (citrate + ATP → acetyl–CoA + oxaloacetate) or pyruvate formate lyase (pyruvate → acetyl–CoA + formate); both of which has previously been shown to boost lipid accumulation (Xu et al., 2016). Overexpression of native *Y. lipolytica* ATP-citrate lyase resulted in modest improvements; however, heterologous ATP-citrate lyases might be proven more beneficial. Organic acid secretion might also be decreased by increasing the indirect pull from enzymes further downstream in the pathway (**Figure 2**). Overexpression of acetyl–CoA carboxylase (*ACC1*) and stearoyl–CoA desaturase (*SGD1*) in combination with diacylglyceride acyl-transferase (*DGA1*) has been shown to greatly boost lipid accumulation in *Y. lipolytica* (Qiao et al., 2015). In terms of fatty alcohol production, it’s possible that the same strategy could be implemented, but replacing *DGA1* with *FAR*.

This study describes a multi-omics analysis of the cellular and metabolic response to fatty alcohol production in two yeasts. It revealed cell wall stress response in fatty alcohol–producing *S. cerevisiae*. Furthermore, we have suggested designs that might aid in the engineering of fatty alcohol–producing cell factories.

## Data Availability

The datasets generated for this study can be found in European Nucleotide Archive, PRJEB32352.

## Author Contributions

IB, JD, and CH conceived the study. JD performed the experiments. EM, CH, GW, and IB aided in troubleshooting and data interpretation. JD and DM performed method development, sampling and data processing of metabolomics data. JD and DW performed sample preparation and GCMS analysis for ^13^C-flux analysis. JD and UL performed data analysis of ^13^C-flux analysis data with the aid of BE. JD and CL performed data analysis of transcriptomic data. GCMS analysis of fatty alcohols was performed by JD, with the aid of H-LW. JD and IB wrote the manuscript with support from CH, EM, GW, UL, BE, MH, and LB. IB, LB, MH, and BE supervised the project. CH and GW helped supervise the project.

## Funding

This project has received funding from the European Union’s Horizon 2020 research and innovation programme under the Marie Skłodowska-Curie grant agreement No 722287. IB acknowledges the financial support from the Novo Nordisk Foundation (Grant agreement NNF15OC0016592 and NNF10CC1016517) and from the European Research Council under the European Union’s Horizon 2020 research and innovation programme (YEAST-TRANS project, Grant Agreement No 757384). MH, CL, and IB acknowledge the received funding from the European Union’s Horizon 2020 research and innovation programme under grant agreement No. 760798 (OLEFINE project). LMB acknowledges funding by the Cluster of Excellence “The Fuel Science Center – Adaptive Conversion Systems for Renewable Energy and Carbon Sources,” which is funded by the Excellence Initiative of the German federal and state governments to promote science and research at German universities.

## Conflict of Interest Statement

IB and CH have a financial interest in BioPhero ApS. The remaining authors declare that the research was conducted in the absence of any commercial or financial relationships that could be construed as a potential conflict of interest.
