# Corrigendum: Multi-Omics Analysis of Fatty Alcohol Production in Engineered Yeasts *Saccharomyces cerevisiae* and *Yarrowia lipolytica*

**DOI:** 10.3389/fgene.2020.637738

**Published:** 2021-01-11

**Authors:** Jonathan Dahlin, Carina Holkenbrink, Eko Roy Marella, Guokun Wang, Ulf Liebal, Christian Lieven, Dieter Weber, Douglas McCloskey, Hong-Lei Wang, Birgitta E. Ebert, Markus J. Herrgård, Lars Mathias Blank, Irina Borodina

**Affiliations:** ^1^The Novo Nordisk Foundation Center for Biosustainability, Technical University of Denmark, Kongens Lyngby, Denmark; ^2^iAMB – Institute of Applied Microbiology, ABBt – Aachen Biology and Biotechnology, RWTH Aachen University, Aachen, Germany; ^3^Department of Biology, Lund University, Lund, Sweden

**Keywords:** fatty alcohol, metabolome, ^13^C-fluxome, transcriptome, *Yarrowia lipolytica*, *Saccharomyces cerevisiae*

Hong-Lei Wang was not included as author in the published article. The corrected Author Contributions Statement appears below.

**Corrected Author Contributions Statement:**

“IB, JD, and CH conceived the study. JD performed the experiments. EM, CH, GW, and IB aided in troubleshooting and data interpretation. JD and DM performed method development, sampling and data processing of metabolomics data. JD and DW performed sample preparation and GCMS analysis for ^13^C-flux analysis. JD and UL performed data analysis of ^13^C-flux analysis data with the aid of BE. JD and CL performed data analysis of transcriptomic data. GCMS analysis of fatty alcohols was performed by JD, with the aid of H-LW. JD and IB wrote the manuscript with support from CH, EM, GW, UL, BE, MH, and LB. IB, LB, MH, and BE supervised the project. CH and GW helped supervise the project.”

In the original article, there was an error in regards to a temperature setting in the fatty alcohol GCMS method. The original article says “The injector was set to splitless mode at *220*°*C*,…”, but should say “The injector was set to splitless mode at 250°C,…”.

A correction has been made to Section “**Materials and Methods**”, subsection “**Fatty alcohol extraction**”, Paragraph 2:

“The analysis was carried out on a GC-MS using an INNOWax column (30 m × 0.25 mm × 0.25 μm) with helium as carrier gas. The injector was set to splitless mode at 250°C; the oven temperature was set to 80°C for 1 min, increased at a rate of 10°C/min to 210°C, followed by a hold at 210°C for 15 min, increased at a rate of 10°C/min to 230°C followed by a hold at 230°C for 20 min. The GC-MS was operated in electron impact mode (70 eV), scanning at the range 30–400 m/z. Compounds were quantified relative to the internal standard (methyl cis-10-heptadecenoate).”

Additionally, in the original article, there was an error in the acknowledgment section, as the acknowledgment of Prof. Christer Löfstedt had been neglected.

A correction has been made to “Acknowledgments”.

“We thank Alexandra Hoffmeyer and Pannipa Pornpitakpong, for performing the RNA-seq analysis. We would also like to thank Dr. Hanne Bjerre Christensen and Dr. Lars Schrübbers for LC-MS and GC-MS analysis. We thank Prof. Christer Löfstedt (Lund University) for analytical advice and access to GC-MS equipment for fatty alcohol analysis. We thank Prof. Volker Zickermann (Goethe-Universität) for the gift of *Y. lipolytica* GB20 strain and Dr. Peter Kötter, Johann Wolfgang Goethe University, Frankfurt, Germany for the gift of the *S. cerevisiae* CEN.PK113-7D strain. We would also like to thank Suresh Sudarsan and Marie Inger Dam for fruitful discussions.”

The authors apologize for this error and state that this does not change the scientific conclusions of the article in any way. The original article has been updated.

